# Three-Dimensionally Hierarchical Graphene Based Aerogel Encapsulated Sulfur as Cathode for Lithium/Sulfur Batteries

**DOI:** 10.3390/nano8020069

**Published:** 2018-01-26

**Authors:** Haipeng Li, Liancheng Sun, Zhuo Wang, Yongguang Zhang, Taizhe Tan, Gongkai Wang, Zhumabay Bakenov

**Affiliations:** 1 School of Materials Science & Engineering, Research Institute for Energy Equipment Materials, Hebei University of Technology, Tianjin 300130, China; lihp_hebut@outlook.com (H.L.); lcsun1992@hotmail.com (L.S.); zhuowang1992@hotmail.com (Z.W.); 2 Synergy Innovation Institute of GDUT, Heyuan 517000, China; taizhetan@gdut.edu.cn; 3 National Laboratory Astana, Nazarbayev University, Institute of Batteries LLC, 53 Kabanbay Batyr Avenue, Astana 010000, Kazakhstan; zbakenov@nu.edu.kz

**Keywords:** lithium/sulfur battery, cathode, sulfur/activated carbon/graphene aerogel composite, reduced graphene oxide, electrochemical performance

## Abstract

A simple and effective method was developed to obtain the electrode for lithium/sulfur (Li/S) batteries with high specific capacity and cycling durability via adopting an interconnected sulfur/activated carbon/graphene (reduced graphene oxide) aerogel (S/AC/GA) cathode architecture. The AC/GA composite with a well-defined interconnected conductive network was prepared by a reduction-induced self-assembly process, which allows for obtaining compact and porous structures. During this process, reduced graphene oxide (RGO) was formed, and due to the presence of oxygen-containing functional groups on its surface, it not only improves the electronic conductivity of the cathode but also effectively inhibits the polysulfides dissolution and shuttle. The introduced activated carbon allowed for lateral and vertical connection between individual graphene sheets, completing the formation of a stable three-dimensionally (3D) interconnected graphene framework. Moreover, a high specific surface area and 3D interconnected porous structure efficiently hosts a higher amount of active sulfur material, about 65 wt %. The designed S/AC/GA composite electrodes deliver an initial capacity of 1159 mAh g^−1^ at 0.1 C and can retain a capacity of 765 mAh g^−1^ after 100 cycles in potential range from 1 V to 3 V.

## 1. Introduction

Critical depletion of energy sources, environmental problems and climate change issues have demanded and encouraged the development of renewable and ecological sources of energy such as wind, solar, etc. However, the intermittent and unstable nature of such resources requires their stabilization and mitigation of fluctuation before integrating into the electrical grids. Such needs, consequently, urgently require the development of high-capacity, reliable and clean energy storage systems [1,2,3]. The rapid growth of ecological electric transport market demands the development of such batteries as well. Currently, lithium-ion batteries (LIBs) are dominating the market of batteries for these applications. However, insufficient power and energy densities, high cost and the use of toxic materials in LIBs restrict them from meeting the requirement for next generation energy-storage systems. In this respect, lithium/sulfur (Li/S) batteries with sulfur cathode have attracted vast attentions worldwide due to their high theoretical specific capacity (1672 mAh g^−1^) and specific energy (2600 Wh kg^−1^) [4]. In addition, sulfur is abundant in the nature and widely available, e.g., produced as a side-product in oil and gas industry, low cost, and non-toxic, providing the Li/S batteries a great potential to be the best candidate for next generation high-energy density storage system [5]. Despite these promising advantages, commercial application of these batteries is still impeded by some critical problems. A low electronic conductivity (5 × 10^−30^ S cm^−1^ at 25 °C) and inferior cycle performance of pure sulfur lead to a low specific capacity and poor utilization of active material. Furthermore, solubility of intermediate products of electrochemical processes of sulfur cathode, polysulfides, into electrolyte during cycling results in a rapid capacity fading and reducing its coulombic efficiency. Finally, a large volume expansion of sulfur cathode upon lithiation further degrades its cycle stability. A combined effect of these processes has severely limited practical application of Li/S batteries [6,7,8].

Various strategies have been proposed to overcome the aforementioned challenges and to ameliorate the electrical conductivity of sulfur cathode and reduce the dissolution and shuttle of polysulfides. Dispersing sulfur into various carbon matrices such as graphene, carbon nanotubes, carbon nanofibers, carbon spheres, etc. is particularly attractive to prepare sulfur-based composite electrodes with enhanced performance [7,9,10,11,12]. Among these carbon materials, graphene and reduced graphene oxide are especially promising for these purposes due to their abilities to prevent the polysulfides diffusion and improve conductivity of sulfur cathode. It is believed that the 3D interconnected graphene framework with high specific area can also allow for an increased sulfur loading into the electrode and, consequently, can increase utilization of active material [13]. In addition, functionalized graphene (reduced graphene oxide) with oxygen containing groups also helps to confine sulfur and restrict diffusion of polysulfides enhancing the electrochemical performance of Li/S batteries [14]. Unfortunately, graphene sheets with two-dimensionally flat surfaces tends to aggregate, decreasing the accessible surface area and limiting the pathways for transmission of Li ions and electrons. This leads to a severe polysulfides diffusion away from the graphene structure in the organic electrolyte and significantly undermines the cycling stability of a cell. Meanwhile, combining the graphene aerogels with activated carbons leads to formation of a stable 3D-network, which can effectively mitigate agglomeration of graphene nanosheets [14,15]. Such incorporated porous structure facilitates the ionic and electronic transport. Moreover, the 3D interconnected structure can also provide space to accommodate more sulfur and enhance the ion transfer during charge/discharge process, enhancing rate capability of the electrode. The graphene-coated carbon-sulfur composites have displayed high specific capacity and cyclic stability as a cathode for Li/S batteries.

In this study, we introduce an effective synthetic method to synthesize a novel sulfur/activated carbon/graphene aerogel (S/AC/GA) composite via a facile reduction of graphene oxide (GO) with developed and highly stable 3D-network structure composed of interconnected graphene sheets and activated carbon. Along with this, we present verification of fundamental characteristics of the prepared composite based on systematic electrochemical studies. The S/AC/GA composite exhibited a high initial capacity and stable cycling performance as a cathode for Li/S batteries, demonstrating the benefits of micro/mesoporous structure and reduced graphene oxide sheets with oxygen groups for trapping sulfur and polysulfides. 

## 2. Materials and Methods

### 2.1. Material Preparation

Graphene oxide was prepared from natural flake graphite by a modified Hummer’s method. A quantity of 3 mg acid-treated activated carbon and 1.5 mL of GO suspension (2 mg mL^−1^) were mixed and ultrasonically dispersed at room temperature for 10 min. After this, a drop of sodium L-ascorbat was added into the AC/GO supernatant fluid (1 mL) and the solution was kept in oil bath at 100 °C for 2 h to form AC/GA. The resulting AC/GA hydrogel was freeze-dried for 12 h at −45 °C and a reduced pressure of 20 Pa.

10 mg of the AC/GA composite and 20 mg of sulfur were thoroughly mixed by ball-milling for 2 h at 300 r min^−1^. The S/AC/GA composite was obtained by drying the resulting mixture at 60 °C in a vacuum oven for 10 h, followed by heating at 155 °C in Ar for 10 h to promote sulfur penetration into the AC/GA pores via vapor phase infusion. The S/AC and S/GA composites were prepared for comparison purposes using the same procedures. The nano-sulfur powder was used as received (MS-P100, Shanghai Huzheng Nano Technology Co., Ltd., Shanghai, China).

The particle size of activated carbon was within 0.4–1.0 µm, the calculated specific surface area of activated carbon was 1508.4 m^2^ g^−1^ and the corresponding pore size distribution was 0.239 cm^3^ g^−1^. 

### 2.2. Structural Characterization

The crystalline structure of the as-prepared samples was investigated by X-ray diffraction (XRD, Smart Lab, Rigaku Co., Tokyo, Japan) equipped with Cu Kα radiation at room temperature. The Raman spectra were recorded on Lab RAM HR800 (Horiba, Tokyo, Japan) using 632 nm laser excitation. The S content in S/AC/GA composite were estimated using a thermogravimetric analyzer (TGA, STD Q-600, TA Instruments-Waters LLC, Newcastle, PA, USA) in N_2_ flow (30 mL min^−1^). Brunauer-Emmett-Teller (BET) (V-Sorb 2800P, Gold APP Instrument Corporation China, Beijing, China) tests were performed to analyze the specific surface and porosity of the composites. Scanning electron microscopy (SEM, S-4800, Hitachi Limited, Tokyo, Japan) and transmission electron microscopy (TEM, JEM2010F, JEOL, Tokyo, Japan) were adopted to observe morphology and microstructure of the samples.

### 2.3. Electrochemical Analysis

Coin-type cells (CR2025) were used to investigate electrochemical properties of the composites. The working electrode was prepared by mixing the active material (the composites), carbon black (AC) and a binder (polyvinylidene fluoride, PVdF) in *N*-methyl-2-pyrrolidone (NMP) in a mass ratio of 8:1:1. The obtained slurry was coated on aluminum foil, which was then dried at 55 °C for 10 h in a vacuum oven. The working electrode was manufactured by punching Al foil with coated electrode into the disks with a diameter of 1.5 cm and pressing them by a hydraulic press (YP-15, Tianjin Henchid Science Technology Development Co., Ltd., Tianjin, China) at 10 MPa. The coin cells were assembled in an argon-filled glove box (99.9995% purity, LABmaster Pro, MBRAUN, Garching, Germany), using pure lithium metal foil as a counter and reference electrode, microporous polypropylene as a separator, and 1 M lithium trifluoromethanesulfonate (LiCF_3_SO_4_) in dimethoxy ethane (DME) and 1,3-dioxolane (DOL) (1:1 *v*/*v*) as an electrolyte. Discharge/charge and cycle life tests of the S/AC/GA composite were conducted between 1.0–3.0 V vs. Li/Li^+^ using a Neware battery tester (BTS 4000, Neware Inc., Shenzhen, China). The cyclic voltammetry (CV) was conducted at a scan rate of 0.1 mV s^−1^ in a potential range of 1.0–3.0 V vs. Li/Li^+^, and the electrochemical impedance spectra were recorded within a frequency range of 10 kHz–10 mHz using an electrochemical workstation (Princeton Applied Research, Versa STAT4, Ametek, PA, USA). The sulfur loading was approximately 3.12 mg cm^−2^ per an electrode circle of 1.5 cm in diameter. The sulfur content was defined based on the total mass of the composites. The sulfur content in the reference S/AC and S/GA electrodes was the same as in S/AC/GA. All experiments were carried out at room temperature. 

## 3. Results and Discussion

**Figure 1:**
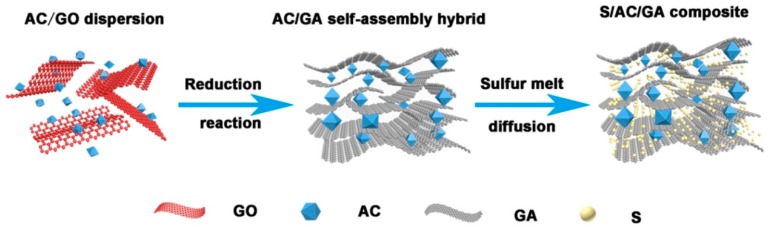
Schematic diagram of fabrication of sulfur/activated carbon/graphene (reduced graphene oxide) aerogel (S/AC/GA) composite.

The preparation procedures of the composites are illustrated in Figure 1. The GO and AC were first mixed ultrasonically in deionized water to form a homogeneous dispersion. Then the AC/GA aerogel was synthesized via a reduction induced self-assembly process with introduction of AC into the graphene aerogels. The pore volume of the composite increased due to presence of activated carbons, which created more surface area accessible for electrochemical interactions. Further, sulfur was homogeneously dispersed in the AC/GA composite via heat treatment and vapor phase infusion method, ensuring its good contact with the conductive substrate.

**Figure 2:**
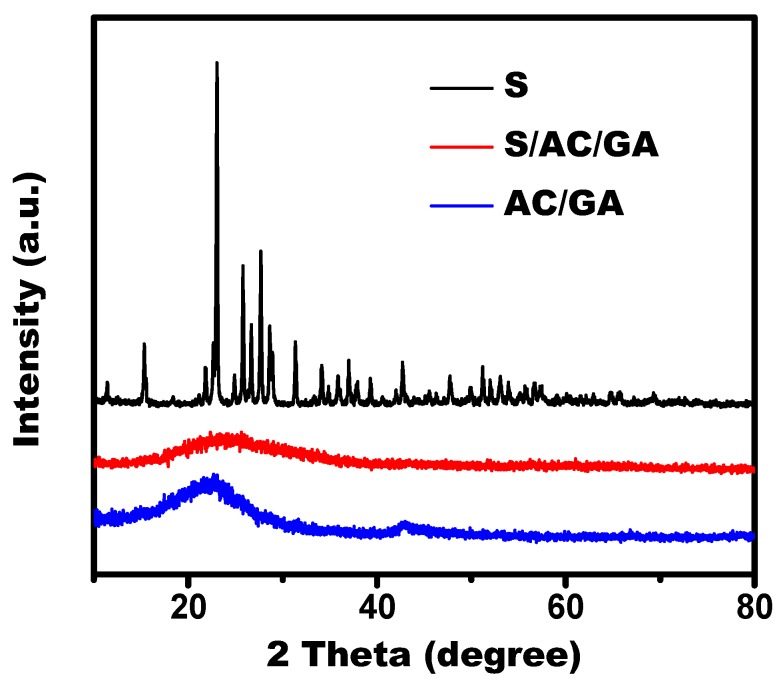
X-ray diffraction (XRD) patterns of elemental sulfur, AC/GA and S/AC/GA composites.

Structural characterization and composition of the samples were observed by XRD and Raman, respectively. Figure 2 shows the XRD pattern of elemental sulfur, the AC/GA as well as S/AC/GA composites. The as-prepared AC/GA composite shows the main characteristic diffraction peaks around 24.6° corresponding to the (002) diffraction plane of graphite, which has a special layer-by-layer structure [16]. In addition, the AC/GA composite shows a typical peak at about 43.2° of the (101) diffraction of graphite. A strong and sharp peak of bulk sulfur in these spectra was identical to the PDF (Powder Diffraction Files) No. 74-1465 of S_8_ molecules standard, whereas this peaks was not observed in the S/AC/GA composite XRD patterns, suggesting that sulfur was well dispersed in the pores of this composite [17].

**Figure 3:**
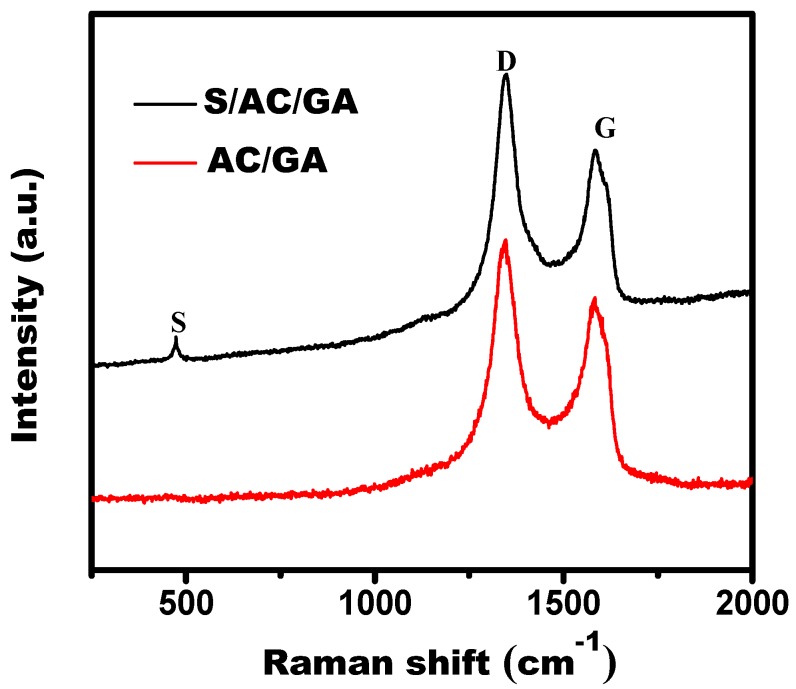
Raman spectra of AC/GA and S/AC/GA composites.

Figure 3 presents the Raman spectra of the AC/GA and the S/AC/GA composites, which shows two remarkable peaks at around 1348 cm^−1^ and 1584 cm^−1^, corresponding to the D-band and G-band of multilayered graphene [16]. The G band represents the vibration of the *E*_2g_ stretching mode of C sp^2^ atoms, meanwhile the D band indicates dispersive defects and disorders. In general, the intensity ratio of D and G peak (*I*_D_/*I*_G_) reflects the degree of graphitization and relative motion of the C sp^2^ atoms. As shown in Figure 2, the *I*_D_/*I*_G_ value of the S/AC/GA composite is 1.44, indicating that GO was efficiently reduced by sodium L-ascorbate [18]. The structure of edges and the unrepaired defects after doping with sulfur promote the electrical conductivity of the S/AC/GA composites, positively affecting its performance in Li/S battery.

**Figure 4:**
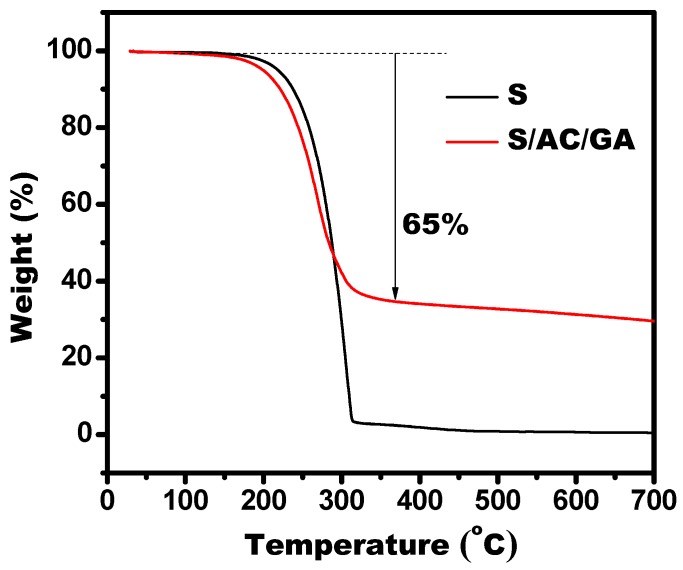
Thermogravimetric analysis (TGA) data of S/AC/GA composite.

Figure 4 displays the TGA curves of S and S/AC/GA. The S/AC/GA composite loses about 65% of its weight at 200 °C, which is due to evaporation of sulfur. Achieving such a high sulfur content in the S/AC/GA composite could be possible due to benefits of its porous structure. The ratio of AC/GA composite and sulfur equal to 1:2 can satisfy the requirements for sulfur content in the cathode to enable achieving a high capacity performance. Furthermore, this value corresponds to the ratio of the components used to prepare the composite, i.e., the sulfur loss upon preparation was minimal. It can be concluded that the 3D hierarchical porous structure of the as-prepared S/AC/GA composite is beneficial for increased sulfur content in the electrode and, consequently, for enhanced energy storage.

**Figure 5:**
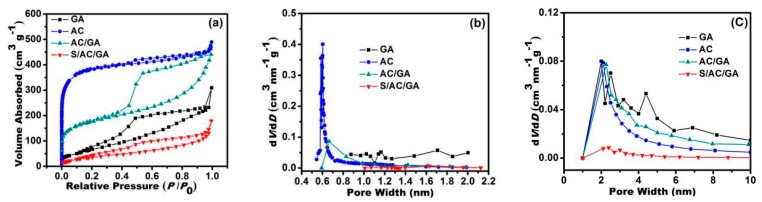
(**a**) N_2_ adsorption/desorption isotherms, (**b**,**c**) Pore size distributions of GA, and AC, AC/GA, and S/AC/GA composites, respectively.

As shown in Figure 5, the porous microstructure and pore size distribution of the GA, AC, AC/GA and S/AC/GA composites was studied and confirmed through their adsorption/desorption isotherms. According to the International Union of Pure and Applied Chemistry (IUPAC) classification, the unmixed GA shows the type II isotherms together with some features of the type IV, indicating a very limited micro and mesoporosity in GA. The modified activated carbon shows a typical type IV isotherm and its adsorption increases sharply at a relatively low pressure, indicating a large number of micropores in its structure. In the case of AC/GA, a typical mesoporous hysteresis loop of type H4 could be observed at a very low relative pressure, reflecting presence of hierarchical porous structure mainly composed of micro and mesopores [19]. Specific surface area values for the AC, GA, AC/GA, and the S/AC/GA composites, calculated by the BET method, were about 1508.4, 260.2, 638.5, and 133.0 m^2^ g^−1^, respectively. After sulfur loading, the specific surface area of AC/GA composite decreased significantly, indicating impregnation of a large amount of sulfur into its pores and surfaces, which corresponds with the TGA data above. The details of corresponding pore size distribution and pore volumes are shown in Figure 5b,c. The mesopores distribution was evaluated based on the BJH-adsorption pore area distribution and the micropore distribution was determined based on the SF-pore size distribution data. GA exhibits a limited proportion of micropores (<2 nm in size) and small size mesopores, while activated carbon shows a narrow pore size distribution around 1–2 nm and mesopores. The AC/GA composite shows a mixture of features of AC and GA, i.e., a high content of micropores along with fewer mesopores, which is consistent with the nitrogen adsorption/desorption isotherms indications above. This unique porous architecture improves utilization of sulfur and restrains the shuttle effect [15].

**Figure 6:**
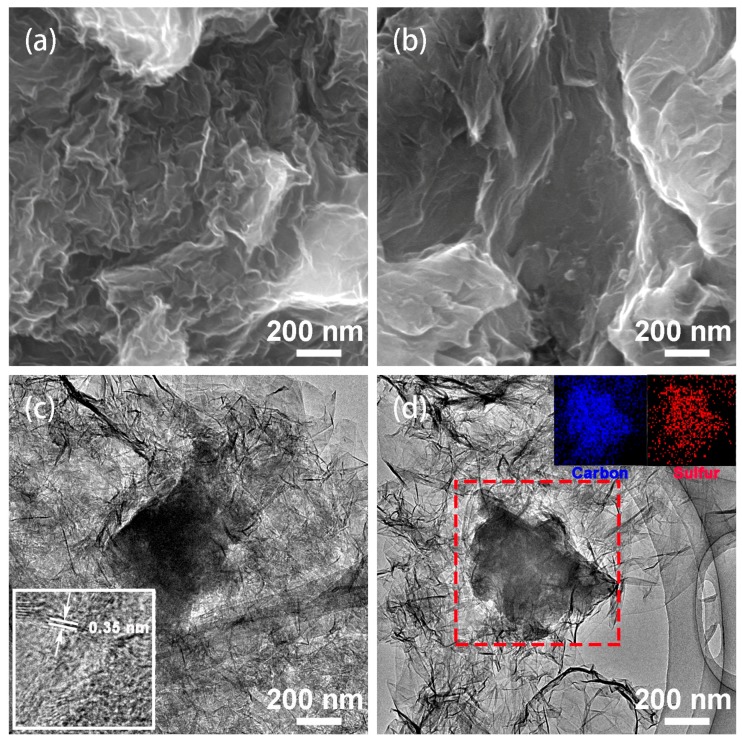
SEM images of AC/GA (**a**) and S/AC/GA composites (**b**), and transmission electron microscopy (TEM) images of AC/GA (**c**) and S/AC/GA composites (**d**); Inset of (**c**) is high-resolution transmission electron microscopy (HRTEM) of S/AC/GA composite; Insets of (**d**) is elemental (carbon, sulfur) mapping of S/AC/GA composite.

The morphology and structure of the as-prepared composites were observed by SEM and TEM. As shown in Figure 6a,b, the as-prepared AC/GA and S/AC/GA composites reveal a sheet-like structure with highly crinkled graphene nano-layer mingled together, while the AC was wrapped uniformly in the AC/GA and the S/AC/GA composites. It can be seen from Figure 6b, that no obvious bulk sulfur can be seen in the image, which supports a suggestion that sulfur was well-distributed in the 3D hierarchical graphene structure, and corroborates with the XRD data [14]. The edge observation of the graphene sheets provides an accurate way to determine the number of its layers. A high-resolution transmission electron microscopy (HRTEM) image (inset of Figure 6c) reveals that graphene possess 2–6 layers with adjacent interlayer spacing of 0.3–0.4 nm. The TEM images of the S/AC/GA composite shows that the samples were mainly composed of a few layers of graphene with AC anchored to the resulting 3D interconnected structure. The carbon and sulfur elemental mappings (inset of Figure 6d) confirms the uniformity of sulfur distribution in the graphene nano-layers [20]. It is worth noting that the crumpled structure was beneficial for sulfur storage and enhanced electronic conductivity of graphene nano-sheets [14]. Meanwhile, the introduced activated carbon not only prevents the graphene sheets from restacking, but also maintains the cross-linked structure and limits the shrinkage of the graphene aerogels [14,15].

**Figure 7:**
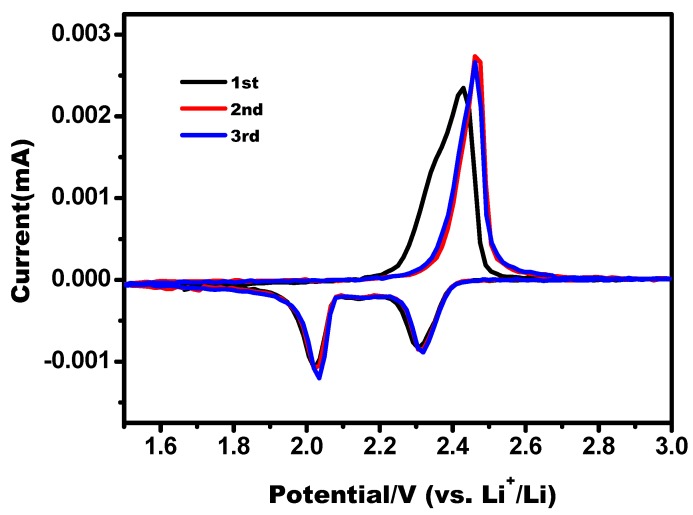
Cyclic voltammograms of S/AC/GA composites at a scan rate of 0.1 mV s^−1^.

Figure 7 shows the CV curves of the S/AC/GA composite at a scan rate of 0.1 mV s^−1^. Two main cathodic peaks at ~2.3 V and ~2.1 V and an obvious anodic peak at ~2.3 V correspond to reversible reduction and oxidation of sulfur, respectively. A reduction peak at 2.3 V is related to conversion of S_8_ to soluble higher-order lithium polysulfide species (Li_2_S*_n_*, 4 ≤ *n* ≤ 8) during the discharge process [20,21]. The next peak at ~2.1 V could be attributed to the further reduction of lithium polysulfides into insoluble Li_2_S_2_ and Li_2_S on the surface of the cathode [6,16]. An oxidation peak at ~2.3 V is ascribed to conversion of Li_2_S_2_ and Li_2_S into lithium polysulfides, and further to elemental sulfur. No remarkable changes in the shape and position of the redox peaks can be detected in the initial three cycles, reflecting a high electrochemical stability and reversibility of the S/AC/GA composite [22].

**Figure 8:**
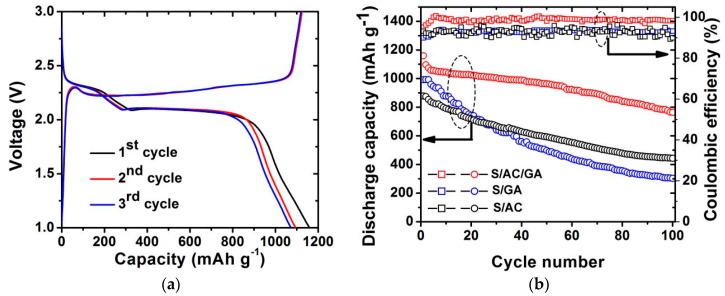
(**a**) Discharge/charge profiles of cell with S/AC/GA composite electrode at 0.1 C; (**b**) Cycle performance of S/AC, S/GA, and S/AC/GA composite electrodes within a potential range from 1.0 V to 3.0 V at a current rate of 0.1 C.

Figure 8a depicts the galvanostatic discharge/charge potential profiles of the S/AC/GA composite at 0.1 C. Two potential plateaus at around 2.3 V and 2.0 V could be observed, which are inherent to Li/S batteries, reflecting the electrochemical stability of the studied system. The discharge/charge curves of the second and third cycles are consistent with each other, which agrees with the CV data above. The higher discharge plateau represents transformation of elemental sulfur into the high-order polysulfides (Li_2_S*_x_*, 4≤ *x* ≤ 8) and the lower discharge plateau represents their further conversion to the low-order polysulfides (Li_2_S_2_/Li_2_S) [23,24,25]. The reverse reactions, corresponding to oxidation of the low-order and high-order polysulfides, are reflected by a single plateau in the charge curves, which also corresponds with the CV results. Figure 8b shows the cycling performance of the S/AC, S/GA, S/AC/GA composites at 0.1 C rate. The S/AC/GA composite delivers an outstanding reversible discharge capacity of 1159 mAh g^−1^ at the first cycle, and the capacity remains as high as 765 mAh g^−1^ after 100 cycles. This enhanced cycling stability is ascribed to the structure and composition of the AC/GA matrix because they were able to effectively conduct electrons and retard the polysulfides diffusion. As for the reference samples, the S/GA and S/AC composites show a relatively low initial discharge capacity of 992 mAh g^−1^ and 877 mAh g^−1^, and these samples could maintain a capacity of only 303 mAh g^−1^ and 443 mAh g^−1^ after 100 cycles, respectively. Due to the abundant micro/mesopores structure, the S/AC composite has a relatively good electrochemical stability compared with the S/GA sample. However, a lower conductivity of the S/AC composite compared with the S/GA composites restricts the active material (sulfur) utilization in it, resulting in a lower reversible discharge capacity compared with S/GA. A high electrical conductivity and 3D-network structure of graphene aerogel effectively improves conductivity of the S/AC/GA composite cathode, and thus provides an enhanced utilization of sulfur. The micro- or mesoporosity of activated carbon facilitates the ion transport and adsorption at the electrode/electrolyte interface, and possesses an optimized packing density of the cell. The AC/GA composite combines the advantages of abundant micro/mesopores structure of AC and high conductivity of GA, which results in excellent electrochemical performance of the S/AC/GA composite.

**Figure 9:**
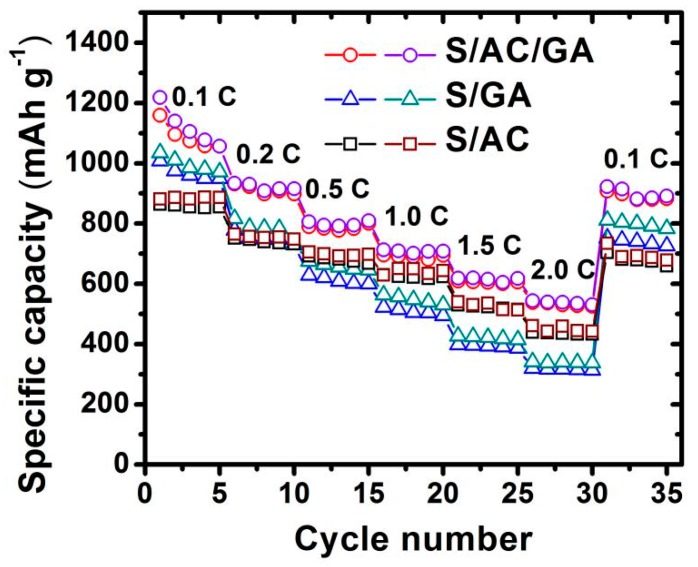
Rate capability of S/AC, S/GA, and S/AC/GA composite electrodes.

The rate capability of the S/AC, S/GA, S/AC/GA cathodes was investigated by cycling at various C rates, as shown in Figure 9. One can see that specific discharge capacity of the S/AC/GA electrode was 1055 mAh g^−1^ at 0.1 C, and it delivers a discharge capacity of 922, 784, 690, 606 and 536 mAh g^−1^ at 0.2, 0.5, 1, 1.5 and 2 C, respectively. Interestingly, it was observed that S/AC/GA exhibits a very steady reversible capacity at each cycling rate from 0.2 C to 2 C, confirming a superior cycling performance of the S/AC/GA cathodes compared with the S/AC and S/GA cathodes. This result is attributed to its stable 3D interconnected AC/GA framework, which provides a larger interfacial/conducting area along with a high electric conductivity of carbon matrix and enhanced Li^+^ ions mass transfer pathways. The reference samples, the S/AC and S/GA cathodes showed inferior rate capability compared with the S/AC/GA cathodes. Among these two samples, a higher discharge capacity was exhibited by S/GA, while a better rate capability was obtained for S/AC cathode, matching well with the cycling performance data in Figure 8b.

**Figure 10:**
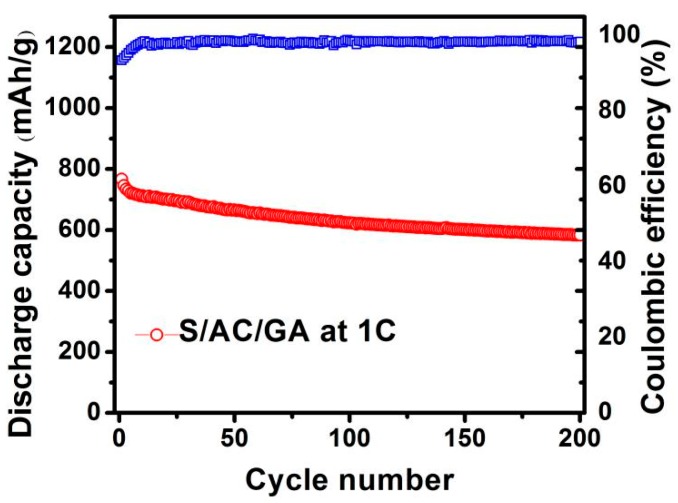
Prolonged cycling performance of S/AC/GA composite electrode at a high rate of 1 C at a potential range of 1–3 V.

Figure 10 shows the long term cycling performance of the S/AC/GA composite at 1 C rate. It can be seen that after 200 cycles at 1 C, the S/AC/GA cathode retains a discharge capacity of 583 mAh g^−1^, which corresponds to a capacity retention of 76.1% and a capacity fading rate of 0.11% per cycle. This indicates the key role of active carbon and graphene, which are confining the polysulfides dissolution into the electrolyte and restricting their shuttling between two electrodes.

**Figure 11:**
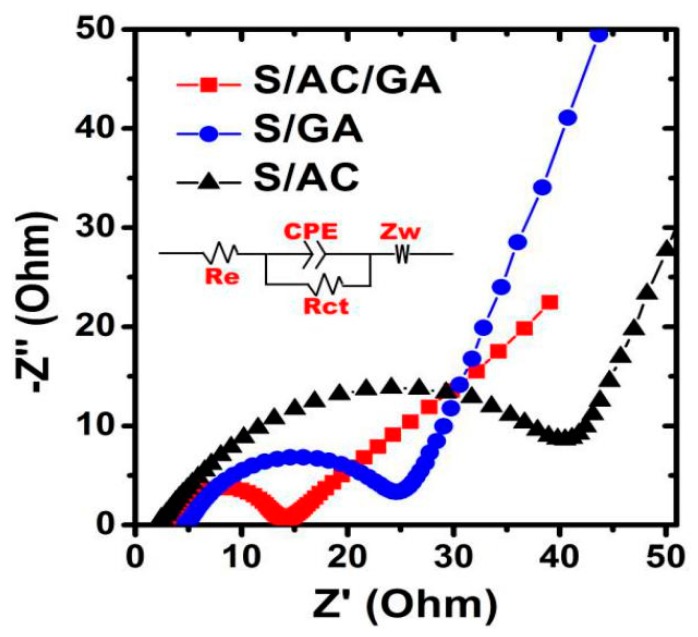
Electrochemical impedance spectroscopy (EIS) impedance spectra of S/AC, S/GA, and S/AC/GA cathodes.

The electrochemical kinetics enhancement of the S/AC/GA electrode can be further confirmed from the electrochemical impedance spectroscopy (EIS) results presented in Figure 11, along with the best fitting equivalent circuit shown in the inset of Figure 11. The circuit consists of an electrolyte resistance (*R*_e_) (a semicircle at high frequency), a charge transfer resistance (*R*_ct_) (a semicircle at medium frequency), and the Warburg resistance (*Z*_w_) (an inclined line in the low frequency region) [26]. The results of the impedance data fitting are shown in Table 1. It can be seen that the *R*_ct_ for S/AC/GA (11.66 Ω) is much lower than that of S/GA (21.34 Ω) and S/AC (35.45 Ω), suggesting a much faster interfacial charge transfer in the S/AC/GA composite than in the S/GA and S/AC composites. 

## 4. Conclusions

In summary, the AC/GA composite was prepared via a reduction induced self-assembly process coupled with the post treatment drying. The AC/GA composite was impregnated with sulfur to obtain the S/AC/GA composite by a simple solution deposition method. The S/AC/GA composite exhibited a high reversible charge/discharge capacity (the initial capacity of 1159 mAh g^−1^) and a stable cycling as along with an excellent rate performance. The enhanced battery performance was assigned to a unique 3D interconnected framework structure of the composite that could effectively entrap the polysulfides and provide fast electron/lithium ion diffusion pathways. These results suggest that the S/AC/GA composite could be considered as a promising cathode for high performance Li/S batteries.

## Figures and Tables

**Table 1:** Equivalent circuit parameters obtained from fitting the impedance spectra.

Electrodes	*R*_e_ (ohm)	*R*_ct_ (ohm)
S/AC/GA	3.57	11.66
S/GA	4.21	21.34
S/AC	3.25	35.45
